# Association between Predictors of Vitamin D Serum Levels and Risk of Retinoblastoma in Children: A Case-Control Study

**DOI:** 10.3390/nu13082510

**Published:** 2021-07-23

**Authors:** Fabiola Mejía-Rodríguez, Mario E. Flores-Aldana, Amado D. Quezada-Sánchez, Teresa Shamah-Levy, Salvador Villalpando, Alejandra Contreras-Manzano, Silvia Bhatt-Carreño, Manuela Orjuela-Grimm

**Affiliations:** 1 Centro de Investigación en Nutrición y Salud, Instituto Nacional de Salud Pública-México, Cuernavaca 62230, Morelos, Mexico; fmejia@insp.mx (F.M.-R.); svillalp@insp.mx (S.V.); alexacont@hotmail.com (A.C.-M.); 2 Centro de Investigación en Evaluación y Encuestas, Instituto Nacional de Salud Pública-México, Cuernavaca 62230, Morelos, Mexico; amado.quezada@insp.mx (A.D.Q.-S.); tshamah@insp.mx (T.S.-L.); 3 Department of Epidemiology, Mailman School of Public Health, Columbia University Medical Center, New York, NY 10032, USA; ssd2120@cumc.columbia.edu (S.B.-C.); mao5@columbia.edu (M.O.-G.); 4 Department of Pediatrics, Division of Oncology, Columbia University Medical Center, New York, NY 10032, USA

**Keywords:** vitamin D, sporadic retinoblastoma, children, women, rural dwelling, ENSANUT

## Abstract

Background: vitamin D (VD) may be a protective factor for retinoblastoma, though no temporal association has been reported during pregnancy or the child’s first year of life. Serum VD concentrations are determined by both distal (DF) and proximal factors (PF). Objective: To identify if DF and PF can predict VD insufficiency (VDI) and VD deficiency (VDD) in women of childbearing age; and to test whether maternal exposure to DF and PF during pregnancy and a child’s exposure during the first 11.9 months postpartum are associated with sporadic retinoblastoma (SRb) in children. Methods: This is a secondary analysis of data from the Epidemiology of SRb in Mexico (EpiRbMx) study and the National Health and Nutrition Survey 2018–2019 (ENSANUT 2018–2019, for its acronym in Spanish). The association of DF and PF with VDD or VDI was estimated using ENSANUT 2018–2019, and the association of DF and PF with SRb using EpiRbMx. All were estimated using logistic regression, with comparable samples selected from ENSANUT 2018–2019 and EpiRbMx. Results: Altitude, latitude and obesity predicted VDI and VDD in ENSANUT women. In EpiRbMx, residence in a rural location during pregnancy increased the risk of SRb. For children, rural residence and latitude increased the risk of SRb, while the number of days exposed to the spring–summer season during months 6 to 11.9 of life was protective. Conclusions: risk of VDI and VDD in women (ENSANUT 2018–2019) increased with altitude, urban dwelling, overweight and obesity. The child and mother’s place of residence, including altitude, latitude and rural classification were important predictors of SRb in EpiRbMx.

## 1. Introduction

Inherited retinoblastoma (Rb) or sporadic retinoblastoma (SRb) develops during pregnancy or infancy [1,2]. The survival rate in low-middle income countries is 70%, while in Mexico it is more than 85%, representing 4.3% of all cases of cancer in children [1,3].

Identifying nutritional determinants of SRb is an intricate task [2,4,5,6,7,8]. In vitro and rodent studies have identified 25OH-vitamin D (VD) as a protective factor for Rb [9,10,11,12]. According to Nebbioso et al. [8], in a systematic review, Vitamin D modulates the immune system, inhibiting inflammation and angiogenesis in eye disorders. Vitamin D may regulate the expression of p21 and p53, thereby inhibiting the growth of retinoblastoma and endothelial cell apoptosis [10,11,12]. VD has also been identified as a protective factor for other types of cancer in children and in adults [13,14,15,16,17,18,19]. The VD synthetized in the skin accounts for 95% of serum concentrations [20,21]. The fetus receives VD through the mother, which increases progressively during the early months of pregnancy and peaks at the third trimester and lowers during lactation [20,21]. However, serum concentrations of VD are determined by distal factors (DF): season of the year, latitude and altitude; and proximal factors (PF): age, skin color, use of sun block, type of clothing, timing of skin exposure to sunlight, intake of supplements, use of micronutrient fortifiers, corporal adiposity, polymorphisms in VD metabolism and retinopathies [20,22,23,24].

In terms of the relationship between retinoblastoma and these factors (DF and PF), one ecological study carried out with children suggests an association between higher altitude and Rb [21]. Other studies have demonstrated that exposure to sunlight decreases the risk of SRb [7,13]. In a cross sectional study, the National Health and Nutrition Survey from 2012 (ENSANUT-2012, for its acronym in Spanish) demonstrated that 36.8% of women had VD deficiency (VDD) and 49.8% insufficiency (VDI) [24]. Because diagnosis of SRb in children is frequently made after infancy (2–6 years of age) and the half-life of serum VD is ~20 days, no temporal association has been reported between the maternal serum concentrations of VD and SRb during pregnancy or the child’s first year of life [1,22,25].

The hypothesis postulated was that DF and PF, which are determinants of VD synthesis, can predict VDD and VDI (serum concentrations of VD). Therefore, we hypothesize that the DF and PF to which the mother was exposed during pregnancy and to which her child was exposed during the first year of life are associated with SRb risk in children. The objective of this study was to identify if DF and PF can predict VDD or VDI in women; and to test whether maternal exposure to DF and PF during pregnancy and the child’s exposure 11.9 months postpartum are associated with SRb in children.

## 2. Materials and Methods

The present study is a secondary analysis of data from the retrospective study “Epidemiology of Sporadic Retinoblastoma in Mexico” (EpiRbMx) [4,5,6,7], including a subgroup of mother-child dyads representing 191 cases and 144 controls. EpiRbMx was designed by the University of Columbia, New York (CUMC), Hospital Infantil de México “Federico Gómez” (HIM) and the Hospital de Pediatría, Centro Médico Nacional Siglo XXI (IMSS) to examine risk factors for SRb during pregnancy and children’s first years of life in central and southern Mexico [4,5,6,7].

### 2.1. Population Selection

#### 2.1.1. Cases

Children diagnosed with unilateral SRb at either HIM or IMSS, younger than 6 years of age, with no family history of Rb, whose mothers consented to participate in the study. Diagnosis was made through ophthalmology and pathology exams and the treatment included enucleation of the affected eye [4,5,6,7].

#### 2.1.2. Controls

Control children were recruited from the same population base, ensuring that the control group was similar to the group of cases. Control children were children of the mothers’ friends; without a cancer or genetic syndrome diagnosis; not biologically related to the case mother (approved by the National Cancer Institute); and of similar age (±1 year) [4,5,6,7].

#### 2.1.3. Exclusion Criteria (Cases and Controls)

To have a family history of retinoblastoma or any other genetic syndrome [4,5,6,7].

Because in EpiRbMx it was not possible to measure of serum VD, in mother during pregnancy or children during first year of life, to identify if the DF and the PF can predict VDD or VDI, we analyzed an independent national representative sample of women at reproductive age (*n* = 1348) ENSANUT 2018–2019. Details of the sub-sample are described below. The methodological details of ENSANUT 2018–2019, sampling characteristics and procedures have been previously described [26]. Briefly, the ENSANUT 2018–2019 was originally designed as a multistage population-based, probabilistic survey with national representativeness by urban and rural settings. Following the analysis of ENSANUT, we identified DF and PF to which pregnant women and their children (during the first 11.9 months) were exposed to in EpiRbMx and examined their association with SRb.

#### 2.1.4. Subsample Selection

As described above, a subsample of women was selected from ENSANUT 2018–2019. To achieve comparable samples between ENSANUT 2018–2019 and EpiRbMx, women were grouped by similarities in schooling, state and municipality of origin, altitude, altitude, longitude, and urban and rural dwelling through a logistic regression model and linear prediction score. Women above the 99th percentile in EpiRbMx and below the 1st percentile in ENSANUT 2018–2019 were excluded in order to eliminate extreme values in both studies.

### 2.2. Data Collection

The sociodemographic and diet questionnaires that were designed and validated for ENSANUT 2018–2019 and EpiRbMx were administered to women from ENSANUT 2018–2019 and mothers from EpiRbMx querying about the mothers exposures during pregnancy and child’s diet during the first 11.9 months of life). Interviews were administered by trained personnel during home visits. Some case mothers from EpiRbMx were interviewed at the HIM or IMSS while their child was receiving care. For EpiRbMx, all procedures were applied, reported and recorded in the same way for both cases and controls [4,5,6,7,26,27,28,29]. The DF and PF from both studies are described together, distinguishing when necessary by ENSANUT 2018–2019 or EpiRbMx.

### 2.3. VD Concentrations from ENSANUT 2018–2019

Fasting blood samples (15 mL) were obtained from the antecubital vein of women (after an 8 h fast) and spun down at 3000 g in situ; serum was placed in criovials of 2 mL, stored in liquid nitrogen (Dewars), and transported to the biochemistry lab of Instituto Nacional de Salud Pública, Cuernavaca, Morelos, Mexico. VD was measured using a chemoluminiscence microparticles immunoassay, (Architect^®^ immunoanalyzer (Abbott Laboratories, MI, III USA) [25,30,31]. The intra- and inter-assay coefficients of variation were 1.34% and 3.69%. Standard Reference Serum NIST 968E from the National Institute of Standards and Technology (100 Bureau Drive, Gaithersburg, MD, USA) was used as quality control of the measurements. Cutoffs for VD were: VDD < 50 nmol/L, VDI ≥ 50–75 nmol/L, sufficiency > 75 nmol/L [25,30,31].

### 2.4. Distal Factors (DF)

The altitude and latitude were calculated from the Instituto Nacional de Estadística, Geografía e Informática database by state, municipality, and place of residence of women or mothers in both studies [32]. For EpiRbMx, the number of days exposed to the spring–summer season during pregnancy was estimated for a period of nine months prior to birth or seven months prior to birth for women with preterm births. For children in EpiRbMx, the number of days exposed to the spring–summer season was calculated from birth to 5 months and from 6 to 11.9 months. The season of the year for EpiRbMx was calculated at the beginning of the first trimester of pregnancy and the month when the offspring reached 6 or 11.9 months of age, considering if it was term or preterm [28]. The season of the year for ENSANUT 2018–2019 was calculated with the date that the blood sample was obtained [33].

### 2.5. Proximal Factors (PF)

Both studies collected age of mother and children (at diagnosis), years of schooling, residential setting was categorized as follows: (urban ≥ 2500 habitants) or rural (<2500 habitants); type of occupation, specifically focusing on those with job tasks carried out outdoors: gardening, agriculture or livestock care as a proxy for sunlight exposure [4,5,6,7,26]. For EpiRbMx, a questionnaire documented children’s sunlight exposure during the first 11.9 months of age, recording weekly sun exposure including the number of days per week, and minutes per day per 6 month time period and whether children were covered with clothes or a blanket while exposed. The methodological details have been previously described [7]. One variable was constructed by multiplying days of the week by the minutes of exposure to sunlight during the first 6, and subsequent 6 months (until 11.9 months of age); children who were covered received a value of zero and uncovered a value of one [7].

### 2.6. Dietary Intake

The daily habitual VD intake (mcg/day) in women ENSANUT 2018–2019 or mothers and children EpiRbMx was estimated through validated semiquantitative food frequency questionnaires (SFFQ) for both studies; more details has been reported elsewhere [26,27,28,29]. For EpiRbMx, this was calculated without separating cases and controls, so data were treated similarly. The daily food frequency intake (portions/day) for each food item was multiplied by the VD content in each standard portion, following the food composition table compiled and updated by Instituto Nacional de Salud Publica (INSP) according to a 2010 regulation that mandated VD fortification of milk, infant formula, and dairy products (NOM-243-SSA1-2010) [34]. Vectors of the diet were run to obtain the VD intake (mcg/day) [35]. The sum of all VD in foods was calculated and was adjusted by energy intake by the residuals method [35]. Two variables were created: (1) the total intake of dietary VD and (2) dietary VD plus the contribution of supplements. Finally, both were categorized into tertiles: I = low, II = medium, III = high [28]. For EpiRbMx, the diet terciles during each trimester of pregnancy and for children during the first 11.9 months are reported. Lactation (exclusive or mixed) was used as an adjusting variable, dividing the months into 5 or less and 6 to 11.9 months [28,29]. Unfortunately only a small sample (*n* = 43) had diet data in ENSANUT 2018–2019 (*n* = 43) and information on supplements was not collected, thus only data on diet were used.

### 2.7. Anthropometry

In EpiRbMx data on anthropometrics was not collected. For ENSANUT 2018–2019, body weight and height were measured by trained personnel according to Lohman and Habicht [26,36]. The height was measured with a stadiometer (Seca model-206, Hamburg, Germany) with a capacity of 220 cm and a precision of 1 mm. Body weight in an electronic balance (Seca model-874, Hamburg, Germany) with capacity of 200 kg and precision of 100 g. Body mass index (BMI) was calculated based on the World Health Organization (WHO) criteria. Because of the small sample size, the category < 18.5 was combined with low and normal BMI into a single category = 18.5–24.9, and overweight = 25–29.9 and obesity ≥ 30 [24,36,37].

### 2.8. Ethics Approval and Consent to Participate

Women consented to voluntary and anonymous participation by signing a consent letter. Both studies have been performed in accordance with the Declaration of Helsinki and approved by Ethics Committee of Instituto Nacional de Salud Pública identification code (IC): 473 (EpiRbMx), IC: 1556 (ENSANUT 2018–2019), FWA: 00015605, and University of Columbia, New York (IRB): AAAB2065 (EpiRbMx), FWA: 00002636.

### 2.9. Statistical Analysis

Means for quantitative variables and proportions for categorical variables were presented; and the differences were estimated by Chi-square or *t*-test. The predictions for VDI and VDD were made through multiple logistic regression models which included occupation (whether outdoors), altitude, latitude, longitude and number of days exposed to the spring–summer season during pregnancy or during the first 11.9 months in children. The association between DF and PF with SRb was calculated through an adjusted logistic regression model using the covariables: pregnancy and child age at 11.9 months of age and show Odds Ratio (OR) and confidence interval (CI95%). Graphics Receiver Operating Characteristics (ROC) expecting a value of ≥0.7. The software Stata, v15.0 (Stata Co., Santa Mónica, SA, USA) was used. The statistical significance was set at *p* < 0.05.

## 3. Results

After subsample selection, we analyzed 126 cases and 102 controls for EpiRbMx and 394 women for ENSANUT 2018–2019. Significant differences (*p* < 0.05) were found between ENSANUT 2018–2019 and EpiRbMx samples for mean years of age of mother and children, years of schooling and altitude (Table 1). Among the variables examined in EpiRbMx s, only age differed between cases and controls (higher in controls) (*p* < 0.05). The proportion of children who were reported to consume VD supplements during their first year were 21% in the controls and 30% in cases (Table 1).

### 3.1. Season of the Year

The VD was collected for ENSANUT 2018–2019 mainly in autumn and winter (70%). In EpiRbMx, the season of the year during pregnancy, delivery, and 6 and 11.9 months of age in offspring was distributed in a similar manner in cases and in controls. For mothers working outdoors, there were significant differences (*p* < 0.05) between cases and controls for EpiRbMx and between women from ENSANUT 2018–2019 (Table 2).

### 3.2. Dietary Intake of VD

**Figure 1:**
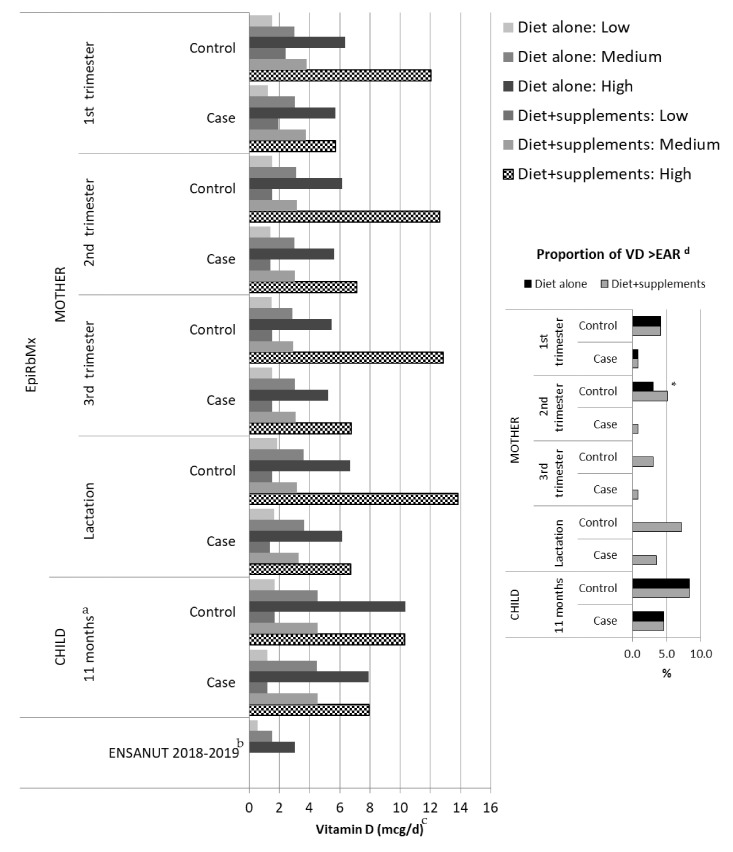
^a^ Mean daily intake of vitamin D (VD) in tertiles, women and children of EpiRbMx and ENSANUT 2018–2019 by evaluation period. ^a^ From starting regular feeding complete diet at 11 months of age, ^b^ no estimation of supplement intake available, ^c^ mean daily intake of vitamin D (ug/day) from diet alone or plus supplements, ^d^ EAR: Estimated Average Requirement, proportion of mother and children EpiRbMx with consumption >10 ug/day of VD (1 to 8 cases), * *p* < 0.05 differences between cases and controls, Case *n* = 115, Control *n* = 97, ENSANUT 2018–2019 *n* = 43.

The proportion of mothers from EpiRbMx who reported consuming VD supplements was similar in both cases and controls. The proportion of mothers with dietary VD intake plus supplements equal or greater than 10 mcg/day was higher (*p* < 0.05) in the second trimester in cases compared with the control group; in control children, the proportion appeared higher than among cases, but differences were not significant (Figure 1).

### 3.3. Prediction of Serum VD Using DF and PF in ENSANUT 2018–2019 Women

The factors predicting risk of VDI in the unadjusted multiple regression model were altitude (OR = 1.08, (CI 95%1.04, 1.12), *p* < 0.001) and rural dwelling (OR = 0.45, (CI 95% 0.23, 0.86), *p* = 0.017). For VDD, the predictive factors were altitude (OR = 1.14, (CI 95% 1.08, 1.21), *p* < 0.0001), latitude (OR = 1.53, (CI 95% 1.02, 2.27), *p* = 0.037) and rural dwelling (OR = 0.33, (CI 95% 0.13, 0.82), *p* = 0.018) (Table 3). The ROC curve gave a value of 0.71 for VDI and 0.84 for VDD; thus, we have moderate discriminatory and predictive capacities for VDI and excellent ones for VDD (data not shown). After adjusting for covariates, the associated factors predicting risk of VDI were altitude (OR = 1.08, (CI 95% 1.03, 1.13), *p* = 0.001), obesity (OR = 446, (CI 95% 1.84, 10.7), *p* = 0.001), and rural dwelling (OR = 0.47, (CI 95% 0.22, 0.99), *p* = 0.049). The risk of VDD was positively associated with latitude (OR = 1.67, (CI 95% 1.10, 2.53), *p* = 0.015), altitude (OR = 1.13, (CI 95% 1.05, 1.20), *p* = 0.001), overweight (OR = 3.73, (CI 95% 1.10, 12.6), *p* = 0.035) and obesity (OR = 6.52, (CI 95% 1.72, 24.5), *p* = 0.006); but negatively associated with rural dwelling (OR = 0.23, (CI 95% 0.07, 0.71), *p* = 0.011) (Table 3). The area under the ROC curve was 0.76 for VDI and 0.87 for VDD, indicating that the model is able to predict better if these variables are included (data not shown).

SRb in the offspring was associated with rural dwelling if the mother lived there during Trimester 1: (OR = 2.65, (CI 95% 1.23, 5.70), *p* = 0.013), Trimester 2: (OR= 2.60, (CI 95% 1.18, 5.68), *p* = 0.017), or Trimester 3: (OR= 2.76, (CI 95% 1.26, 6.0), *p* = 0.011) (Table 4). It was also negatively associated with other supplements in Trimester 2, (OR = 0.46, (CI 95% 0.22, 0.92), *p* = 0.028). The area under the ROC curve for Model I = 0.78, Model II = 0.79 and Model III = 0.78, indicating that the model is able to predict better if these variables are included (Table 4).

### 3.4. Association between Offspring’s DF and PF during <6 Months and 6 to 11 Months of Age and SRb in EpiRbMx

With the variables registered <6 months of age adjusted by all types of lactation, SRb was associated positively with living in a rural dwelling (OR = 2.92, (CI 95% 1.29, 6.55), *p* = 0.010). During 6 to 11 months of age, SRb was positively associated with living in a rural dwelling (OR = 2.94, (CI 95% 1.30, 6.62), *p* = 0.009), (Table 5). With variables measured <6 months of age adjusted by exclusive breast feeding, SRb was marginally and negatively associated with latitude (OR = 0.60, (CI 95% 0.35, 1.02), *p* = 0.060) and positively associated with longitude (OR = 1.50, (CI 95% 1.08, 2.08), *p* = 0.013). During 6 to 11 months, latitude was protective against SRb (OR = 0.87, (CI 95% 0.78, 0.96), *p* = 0.010), as well as the number of days exposed to the spring–summer season (OR = 0.63, (CI 95% 0.41, 0.96), *p* = 0.035); SRb was positively associated with longitude (OR = 1.50, (CI 95% 1.07, 1.04), *p* = 0.018), (Table 5).

## 4. Discussion

### 4.1. Prediction of VDI and VDD Using DF and PF in ENSANUT 2018–2019 Women

Altitude was positively associated with VDI and VDD, and latitude with VDD; nevertheless, for offspring, altitude was not associated with SRb. This is not consistent with some studies that suggest the higher the altitude and the exposure to sunlight, the lower the rate of cancers and mortality [20,22,38,39]. The predictive capacity of VDI (0.68) and VDD (0.81) may be related with the collection date (autumn–winter) or the small sample size of the ENSANUT 2018–2019 diet subsample.

Latitude was significantly associated with VDD; this is consistent with the three bands of environmental UVR identified with enough UVR to produce VD during the whole year (latitude 30° N to 30° S) and the finding that cancer incidence increases with decreasing latitude towards the equator [39,40]. In Mexico, the extreme coordinates are: North: 32°43′06″ latitude north. South: 14°32′27″ latitude north [41].

In ENSANUT-2012, women presented VDD or VDI more frequently if they were classified as overweight or obese and lived in an urban dwelling [24]. In this sample, 24.9% had VDD and 44.7% VDI, and were associated with obesity and living in an urban dwelling.

### 4.2. Association between DF and PF during Pregnancy and SRb in Offspring in EpiRbMx

Living in a rural dwelling during pregnancy was positively associated with SRb, which was consistent with Ramírez-Ortiz et al., but contrary to our association with VDI and VDD [5].

The prevalence of mothers who worked outdoors during pregnancy (which is a proxy of the time exposed to sunlight) was greater in the control group, but differences were not significant; however, it was significantly different to the prevalence in ENSANUT 2018–2019. Mothers in Tertile III (which represents high intake) of VD during pregnancy had an intake of more than 10 μg/day (400 UI/day), but it was not associated with SRb in the offspring. On the other hand, other supplements were protective against SRb [4]. One study in pregnant women proved that an intake of 10 μg/day of VD was insufficient, when exposure to sunlight was scarce; the children then had VDD at delivery [21].

### 4.3. Association between Offspring’s DF and PF during <6 Months and 6 to 11 Months of Age and SRb in EpiRbMx

Latitude and the number of days exposed to the spring–summer season during 6 to 11.9 months of life were negatively associated with SRb in children who had exclusive maternal lactation. A previous analysis of a larger number of cases in EpiRbMx found that exposure to sunlight increased with age; cases were less exposed than controls, but in our analysis of unilateral SRb there were not significant differences [7]. In our analysis, 21.1% of children cases and 30% of controls used supplements, and 8% of controls and 4.3% of cases consumed VD above 10 μg/day, suggesting that the exposure to sunlight was amplified by supplement intake and lactation [20,21,42,43,44].

In terms of limitations, a weakness of this study was the small sample size from ENSANUT 2028-2019 (*n*= 43) of women with dietary information available but no information on supplement intake. Another limitation is the nature of the cross-sectional study design. This is the first study comparing Mexican population-based data on serum VD from ENSANUT 2018–2019 with data from EpiRbMx.

It is also the first study to provide information on mothers who work outdoors during pregnancy and to use this as a proxy of the time exposed to sunlight; additionally, the estimation of number of days exposed to the spring–summer season during pregnancy and during 11.9 months of life children. The study also has several strengths, particularly the data on a large group of women that permitted the prediction of individual IVD and VDD using DF and PF in a comparable samples between ENSANUT 2018–2019 and EpiRbMx. This allowed us to control for both observed and unobserved factors with invariant effects on the outcomes. On the other hand, continuous and rigorous monitoring of recollection of information was performed by highly trained field workers in both studies. As an additional strength, potential reporting and instrument biases are not related to study group by design, allowing us to compare differences between case and control mothers, and between ENSANUT 2018–2019 women. Future research should continue to evaluate VD using DF and PF in pregnant women and children with SRb during their first year of life, particularly the data on high altitude, rural dwelling, overweight-obesity and the time exposed to sunlight.

## 5. Conclusions

In conclusion, in this study we found an increased risk of VDI and VDD with altitude, urban dwelling, overweight and obesity. In addition, we observed an increased risk of SRb in offspring if the mothers lived in a rural dwelling during pregnancy; in children, during 6 to 11.9 months of life, there was a decreased risk of SRb with latitude and the number of days exposed to sun to the spring–summer season. Our data are not conclusive, thus it would be desirable for future analyses to consider a larger sample of women and children, examining risk of sporadic retinoblastoma with greater detail of the mother’s outdoor occupational exposure, maternal hours of sun exposure, use of sunscreen, type of clothing, skin color, weight and height pre-pregnancy, among others. It is recommended that during the first 1000 days of life (intrauterine and at birth), children be exposed to sunlight, especially in rural areas (avoiding midday during the summer); receive VD supplements in non-summer months; and be given appropriate medical care during the first five years of life [45,46].

## Figures and Tables

**Table 1:** Characteristics of women and children of EpiRbMx and ENSANUT 2018–2019.

			EpiRbMx				
		Case		Control		ENSANUT 2018–2019	
Subject of Study	Characteristics	*n*	Mean	*n*	Mean	*n*	Mean
Women	Age (years)	126	25.9	102	26.1	394	29.4 **
	Years of schooling (years)	126	9.9	102	9.2	394	4.4 **
	Altitude (m)	126	17.0	102	17.2	394	12.7 **
	Latitude (grades)	126	19.2	102	19.2	394	19.4
	Longitude (grades)	126	98.7	102	98.6	394	98.8
	Spring–Summer (days) ^a^	126	136.1	102	135.3		
	Vitamin D (nmol/dL)					394	65.8
Children	Age (years) ^b^	126	2.4	102	3.7 *		
	Spring–Summer (days) ^a^	126	3.0	102	3.0		
	Sunshine index (<6 months) ^a^	121	167.3	94	329.4		
	Sunshine index (≥6 months) ^a^	121	457.8	99	408.9		
	Lactation (<6 months) ^c^	41	2.4	37	1.8		
	Lactation (≥6 months) ^c^	32	7.9	26	7.6		
	Maternal lactation (<6 months) ^d^	41	2.9	35	3.2		
	Maternal lactation (≥6 months) ^d^	18	6.3	11	6.0		
**Subject of Study**	**Characteristics**	***n***	**%**	***n***	**%**	***n***	**%**
Women	Serum vitamin D						
	Sufficiency					120	30.5
	Insufficiency					176	44.7
	Deficiency					98	24.9
	Supplements 1st trimester						
	VD	7	6.1	7	7.2		
	others ^e^	55	47.8	52	53.6		
	Supplements 2nd trimester						
	VD	9	7.8	11	11.3		
	Others ^e^	54	47.0	57	58.8		
	Supplements 3rd trimester						
	VD	10	8.7	7	7.2		
	Others ^e^	55	47.8	54	55.7		
	Dwelling						
	Urban	84	66.7	79	77.5	256	65.0
	Rural	42	33.3	23	22.6	138	35.0
Children	Covered <6 months ^a^	37	29.4	26	25.5		
	Covered ≥ 6 months ^a^	8	6.4	6	5.9		
	Supplements ^f^						
	VD	12	21.1	12	30.0		
	Others ^e^	20	35.1	10	25.0		

^a^ During exposure to sunshine, ^b^ at diagnosis ^c^ all type of Lactation, ^d^ exclusive maternal lactation, ^e^ Iron, B12, B6, folic acid, calcium, ^f^ during 11.9 months * *p* < 0.05 differences between case and control, and ** *p* < 0.05 between EpiRbMx and women of ENSANUT 2018–2019.

**Table 2:** Distribution of children and women by season of the year and type of work ENSANUT 2018–2019 and EpiRbMx.

Subject and Study	Period	Season of the Year			
		Spring–Summer		Autumn–Winter	
		*n*	%	*n*	%
ENSANUT 2018–2019 (Women) ^a^	At interview	106	26.9	288	73.1
EpiRbMx (Mother) ^b^	1st trimester				
	Case	110	87.3	16	12.7
	Control	94	92.2	8	7.8
	2nd trimester				
	Case	49	38.9	77	61.1
	Control	41	40.2	61	59.8
	3rd trimester				
	Caso	17	13.5	109	86.5
	Control	10	9.8	92	90.2
EpiRbMx (Child)	At delivery				
	Case	62	49.2	64	50.8
	Control	50	49	52	51
	At 6 months of age				
	Case	64	50.8	62	49.2
	Control	52	51	50	49
	At 11 months of age				
	Case	67	53.2	59	46.8
	Control	51	50	51	50
		**Work Outside Home or Work**			
**Subject and Study**		**Yes**		**No**	
		***n***	**%**	***n***	**%**
ENSANUT 2018–2019 (women)	Any work	159	40.4	235	59.6
	Work outdoors ^c^	24	10.1 **	214	89.9
EpiRbMx (Mother)	First three months ^c^				
	Case	53	43.1	70	56.9
	Control	44	43.1	58	56.9
	Following months ^c^				
	Case	44	36.1	78	63.9
	Control	39	38.6	62	61.4
	Work outdoors ^c^				
	Case	24	19.1	102	81
	Control	25	24.5	77	75.5

^a^ Serum vitamin D date, ^b^ at the beginning of each trimester of pregnancy, ^c^ gardening, agriculture, livestock care, or any other outdoor activity, ** *p* < 0.05 between women ENSANUT 2018–2019.

**Table 3:** Prediction of serum vitamin D using DF and PF determinants in women. ENSANUT 2018–2019.

	VDI ^a^						VDD ^a^					
	Model I ^b^ (*n* = 187 )			Model II ^c^ (*n* = 172)			Model III ^b^ (*n* = 138)			Model IV ^c^ (*n* = 125)		
	OR	CI 95%	*p* Value	OR	CI 95%	*p* Value	OR	CI 95%	*p* Value	OR	CI 95%	*p* Value
Latitude (grades)	1.17	(0.91, 1.48)	0.208	1.23	(0.94, 1.59)	0.120	1.59	(1.07, 2.33)	0.019	1.67	(1.10, 2.53)	0.015
Longitude (grades)	0.90	(0.78, 1.03)	0.140	0.93	(0.80, 1.07)	0.352	0.84	(0.66, 1.05)	0.128	0.88	(0.68, 1.12)	0.303
Altitude (m) ^d^	1.07	(1.03, 1.11)	0.000	1.08	(1.03, 1.13)	0.000	1.13	(1.06, 1.19)	0.000	1.13	(1.05, 1.20)	0.001
Season												
Spring–Summer	ref											
Autumn–Winter	1.56	(0.75, 3.23)	0.235	1.18	(0.51, 2.69)	0.697	1.78	(0.66, 4.76)	0.248	1.83	(0.55, 6.07)	0.320
Outdoor work ^e^												
No	ref											
Yes	1.31	(0.48, 3.56)	0.592	1.41	(0.43, 4.62)	0.569	0.35	(0.05, 2.23)	0.266	0.15	(0.01, 1.63)	0.119
BMI (kg/m^2^)												
Normal BMI	ref											
Overweight				1.95	(0.83, 4.55)	0.122				3.73	(1.10, 12.6)	0.035
Obesity				4.46	(1.78, 11.1)	0.001				6.52	(1.72, 24.5)	0.006
Age mother (years)				0.98	(0.92, 1.04)	0.550				1.02	(0.94, 1.11)	0.564
Dwelling												
Urban	ref											
Rural	0.45	(0.23, 0.86)	0.017	0.47	(0.23, 0.97)	0.041	0.33	(0.13, 0.82)	0.018	0.23	(0.07, 0.71)	0.011
Years of schooling	1.00	(0.87, 1.13)	0.979	1.03	(0.89, 1.18)	0.689	1.05	(0.90, 1.22)	0.529	1.10	(0.90, 1.33)	0.350
Constant	698.89	(0.03, 1320)	0.192	8.59	(0.00, 4431)	0.698	606.53	(0.00, 4220)	0.425	0.41	(0.00, 2790)	0.923

CI 95%: 95% confidence interval, ^a^ VDI: serum vitamin D insufficiency (≥50 and <75 nmol/L), VDD: serum vitamin D deficiency (<50 nmol/L), DF: distal factors, PF: proximal factors. ^b^ Reduced multiple regression model (area under ROC curve I = 0.71 and III = 0.84), ^c^ amplified logistic regression model (area under ROC curve; II = 0.76 and IV = 0.87), ^d^ re-scaled to 100 m, ^e^ gardening, agriculture, livestock care, or any other outdoor activity.

**Table 4:** Association between DF and PF during pregnancy and SRb in offspring in EpiRbMx.

	Model I ^a^			Model II			Model III		
	1st Trimester (*n* = 212)			2nd Trimester (*n* = 212)			3rd Trimester (*n* = 212)		
Risk of Retinoblastoma	OR	CI 95%	*p* Value	OR	CI 95%	*p* Value	OR	CI 95%	*p* Value
Latitude (grades)	0.96	(0.68, 1.33)	0.791	0.97	(0.69, 1.34)	0.839	0.95	(0.68, 1.32)	0.781
Longitude (grades)	1.03	(0.84, 1.25)	0.751	1.02	(0.83, 1.25)	0.811	1.03	(0.84, 1.25)	0.790
Altitude (m) ^b^	0.98	(0.93, 1.02)	0.315	0.98	(0.93, 1.02)	0.320	0.98	(0.93, 1.02)	0.369
Spring–Summer (days)	1.00	(0.99, 1.00)	0.902	1.00	(0.99, 1.00)	0.851	1.00	(0.99, 1.00)	0.862
Outdoor work ^c^									
No	ref								
Yes	0.53	(0.23, 1.17)	0.117	0.54	(0.24, 1.20)	0.134	0.55	(0.25, 1.22)	0.146
Dwelling									
Urban	ref								
Rural	2.65	(1.23, 5.70)	0.013	2.60	(1.18, 5.68)	0.017	2.76	(1.26, 6.00)	0.011
Vitamin D intake (mcg/day) ^d^									
Low	ref								
Medium	0.99	(0.44, 2.17)	0.972	0.96	(0.43, 2.10)	0.915	0.78	(0.35, 1.69)	0.530
High	0.66	(0.29, 1.50)	0.324	0.89	(0.38, 2.03)	0.778	0.73	(0.32, 1.66)	0.456
Supplement									
No	ref								
VD	0.76	(0.21, 2.73)	0.674	0.48	(0.15, 1.48)	0.202	1.23	(0.35, 4.27)	0.744
Other ^e^	0.66	(0.33, 1.28)	0.221	0.46	(0.22, 0.92)	0.028	0.67	(0.34, 1.29)	0.233
Years of Schooling	1.10	(1.00, 1.20)	0.048	1.10	(0.99, 1.20)	0.053	1.10	(1.00, 1.19)	0.045
Age Children (years) ^f^	0.53	(0.42, 0.66)	<0.001	0.53	(0.42, 0.66)	<0.001	0.54	(0.43, 0.66)	<0.001
Constant	0.58	(0.00, 2619)	0.944	1.20	(0.00, 5534)	0.981	0.92	(0.00, 3803)	0.991

DF: distal factors, PF: proximal factors, ^a^ logistic regression model (area under ROC curve; I = 78, II = 0.79 and III = 0.78), OR: odds ratio, CI 95%: 95% confidence interval, ^b^ re-scaled to 100 m. ^c^ Gardening, agriculture, livestock care, or any other outdoor activity, ^d^ vitamin D intake in mcg/day plus supplements, ^e^ iron, B12, B6, folic acid, calcium, ^f^ at diagnosis.

**Table 5:** Association of retinoblastoma with DF and PF during <6 months and 6 to 11 months of age adjusted by variables of interest. EpiRbMx.

		<6 Months						6 to 11 Months					
		Model I ^a,b^ (*n* = 195)			Model I ^a,c^ (*n* = 97)			Model III ^a,b^ (*n* = 195)			Model IV ^a,c^ (*n* = 97)		
Characteristics	Categories	OR	CI 95%	*p* Value	OR	CI 95%	*p* Value	OR	CI 95%	*p* Value	OR	CI 95%	*p* Value
**Latitude (grades)**		0.92	(0.65, 1.29)	0.632	0.60	(0.35, 1.02)	0.060	0.90	(0.63, 1.26)	0.537	0.55	(0.31, 0.93)	0.028
**Longitude (grades)**		1.06	(0.86, 1.30)	0.583	1.50	(1.08, 2.08)	0.013	1.07	(0.87, 1.31)	0.521	1.50	(1.07, 2.10)	0.018
**Altitude (m) ^d^**		0.98	(0.93, 1.02)	0.329	0.95	(0.88, 1.02)	0.190	0.98	(0.93, 1.02)	0.387	0.97	(0.89, 1.04)	0.410
**Outdoors work ^e^**	No	ref											
	Yes	0.55	(0.23, 1.26)	0.159	0.65	(0.19, 2.19)	0.492	0.54	(0.22, 1.25)	0.153	0.66	(0.19, 2.22)	0.508
**Vitamin D intake (mcg/day) ^f^**	Low	ref											
	Medium	0.67	(0.30, 1.50)	0.334	1.02	(0.30, 3.42)	0.981	0.65	(0.28, 1.47)	0.304	1.37	(0.40, 4.65)	0.614
	High	0.93	(0.41, 2.06)	0.855	1.02	(0.31, 3.25)	0.972	0.93	(0.41, 2.06)	0.853	1.07	(0.33, 3.43)	0.912
**Lactation (months)**		1.09	(0.90, 1.31)	0.379	1.03	(0.74, 1.41)	0.869	1.04	(0.95, 1.12)	0.385	1.00	(0.77, 1.30)	0.972
**Covered ^g^**	No	ref											
	Yes	1.27	(0.60, 2.68)	0.528	1.32	(0.42, 4.06)	0.627	1.59	(0.39, 6.45)	0.514	0.89	(0.12, 6.35)	0.906
**Sunshine index**		1.00	(0.99, 1.00)	0.492	1.00	(0.99, 1.00)	0.170	1.00	(0.99, 1.00)	0.124	1.00	(0.99, 1.00)	0.167
**Spring–Summer (days) ^g^**		0.96	(0.76, 1.21)	0.746	0.92	(0.62, 1.34)	0.662	0.84	(0.65, 1.07)	0.161	0.63	(0.41, 0.96)	0.035
Sunshine index # Spring–Summer		1.00	(0.99, 1.00)	0.923	1.00	(0.99, 1.00)	0.183	1.00	(0.99, 1.00)	0.127	1.00	(0.99, 1.00)	0.122
**Dwelling**	Urban	ref											
	Rural	2.92	(1.29, 6.55)	0.010	2.78	(0.85, 9.01)	0.088	2.94	(1.30, 6.62)	0.009	2.52	(0.77, 8.18)	0.125
**Years of schooling**		1.08	(0.98, 1.18)	0.112	1.15	(0.99, 1.33)	0.059	1.08	(0.98, 1.18)	0.103	1.13	(0.97, 1.31)	0.104
**Age Children (years) ^h^**		0.55	(0.44, 0.68)	<0.001	0.66	(0.49, 0.89)	0.008	0.55	(0.44, 0.68)	<0.001	0.66	(0.48, 0.89)	0.007
**Cons**		0.06	(0.00, 4586)	0.731	0.00	(5.39, 0.02)	0.025	0.06	(0.00, 3816)	0.722	0.00	(1.07, 0.62)	0.046

DF: distal factors, PF: proximal factors, ^a^ logistic regression model (area under ROC curve; I = 79, II = 0.78, III = 79 and IV = 0.78), OR: odd ratio, 95% CI: 95% confidence interval, ^b^ all type lactation, ^c^ exclusive maternal lactation, ^d^ re-scalated at 100 m, ^e^ gardening, agriculture, livestock care, or any other outdoor activity, ^f^ vitamin D intake (ug/d) from the diet plus supplements during 11.9 months of age, ^g^ during exposure to sunshine, ^h^ at diagnosis. Association between DF and PF during pregnancy and SRb in offspring in EpiRbMx.

## Data Availability

Manuela Orjuela-Grimm designed the study—EpiRbMx. Teresa Shamah-Levy and Salvador Villalpando designed the study—ENSANUT 2018–2019; all information should be consulted with these authors.
